# Bridging the critical gap between infectious disease blood donation screening and connection to healthcare services: the American Chagas disease example

**DOI:** 10.1057/s41271-024-00539-5

**Published:** 2024-12-18

**Authors:** M. K. Lynn, Mary Parker, Susan L. Stramer, Rebecca L. Townsend, Melissa S. Nolan

**Affiliations:** 1 Institute for Infectious Disease Translational Research, University of South Carolina, Columbia, SC USA; 2 School of Medicine, University of South Carolina, Columbia, SC USA; 3 Independent Infectious Disease Consultant, North Potomac, MD USA; 4 Scientific Affairs, American Red Cross, Gaithersburg, MD USA; 5 Arnold School of Public Health, University of South Carolina, 915 Greene Street, Suite 327, Columbia, SC 29208 USA

**Keywords:** USA, Blood donor screening, Chagas disease, *T. cruzi*, Transfusion-transmitted infections, Rare and neglected infections

## Abstract

Chagas disease (*Trypanosoma cruzi* infection) affects ~ 290,000 USA residents and is included in routine blood donation screening panels. Donors are notified of positive *T. cruzi*-screening results, deferred from donation, and given limited information for next steps. Individuals living with undiagnosed, uncommon infections often face substantial barriers in accessing physicians with infectious disease competency, confirmatory testing, and continuum of care after the point of blood donor deferral. We assessed 46 *T. cruzi*-deferred donors’ experience following deferral, highlight donor challenges, and provide public health institution opportunities to support cases of rare transfusion-transmitted infections in the USA.

## Key messages

- Blood donor screening provides an opportunity to identify potentially rare infections; however, additional clinical guidance is needed for those who screen positive for infections through this national surveillance initiative.
- When rare infections are detected through routine blood donation, many donors face challenges accessing diagnostic-grade testing and quality, informed clinical care. Enhanced support is imperative, but who is responsible and who pays?

## Introduction

Since the 1950s, screening blood products for transfusion-transmitted pathogens has been a mandatory element in the biological sample donation process across the United States of America [1]. The USA Food and Drug administration (FDA) oversees the maintenance and assurance of viable blood products as listed in the Code of Federal Regulations Title 21 parts 610 and 630 [2]. Over time, safeguarding practices have been fortified, and pathogen screening methods have significantly advanced with the advent of new technologies and the identification of additional transfusion-transmitted infections. In addition to blood-based pathogen testing, the FDA maintains quality assurance of blood products through donor screening surveys, deferred donor lists, and standards for handling potentially infectious blood products as well as blood product manufacturing practices [2]. FDA mandated screening of blood products for infectious pathogens began with *Treponema pallidum,* the causative agent of syphilis, in the 1950’s. Since then, the number of infectious diseases screened for in the blood donation process has grown significantly. As of 2022, USA blood centers now screen for HIV, hepatitis B and C viruses, human T-lymphotropic virus (HTLV), *T. pallidum*, West Nile virus, *Babesia spp*. and notably, the subject of this article, *Trypanosoma cruzi*, the causative agent of Chagas disease [3]. While most of the pathogens are considered rare (less than 1 per 100,000 persons), their notable clinical impacts foundationally support their routine screening.

If undiagnosed and untreated, *T. cruzi* can become a chronic, lifelong infection that insidiously causes organomegaly in a subset of infected persons [4]. Following a handful of USA transfusion-acquired cases, universal blood donation screening for *T. cruzi* was implemented in 2007 and later replaced by screening first-time donations [5, 6]. Since it’s inclusion in routine first-time donor screening, this national passive surveillance method has led to the largest number of novel case identifications due to existing challenges with poor USA physician awareness and clinical management competency [7, 8]. Traditionally considered a Latin American endemic disease, ~ 290,000 persons are estimated to be infected the USA, including approximately 10,000 locally acquired cases [9]. Both the *T. cruzi* etiologic agent and triatomine insect vector have been long established in the USA, and autochthonous infections of animal reservoirs and humans have been extensively documented [10–13].

Despite a higher USA disease burden than high profile infections like Zika, Chagas disease is frequently overlooked and underdiagnosed with less than 1% of those infected being diagnosed or treated nationally [14]. Although blood donor testing has provided numerous positive screens, relatively few donors have sought follow-up for diagnostic testing and < 0.3% of estimated USA cases have been ultimately treated [15, 16]. To better understand the critical gap between initial blood donor identification, deferral and subsequent clinical management, this study enrolled donors who screened positive through routine blood donation testing from across the southwestern USA. We present the challenges faced by *T. cruzi* antibody-positive blood donors after the point of blood donor deferral and notification of *T. cruzi* antibody-positive screening results. We call to action for blood centers, hospital systems, and public health institutions to bridge the gap between blood donor screening and clinician managed care.

## Methods

To address the study’s objectives, we conducted structured qualitative interviews to identify key themes and frame the issues leading to lack of clinical management among deferred blood donors. These guided themes were then substantiated using peer-reviewed publications, government regulatory statues, and personal communications with blood center medical directors.

Human subjects’ approval for donor interviews was obtained by the following institutional review boards: the University of South Carolina, The American Red Cross, Vitalant, and Central California Blood Center. As previously described [17], invitation letters were sent by the originating blood donor organization to all *T. cruzi* antibody-positive blood donors from California, New Mexico, Arizona, and western Texas. Interested participants contacted the study team to participate, and 46 individuals were enrolled. The exact recruitment rate was unknown as the originating blood donor organizations likely had outdated addresses on donors deferred several years prior; however, an estimated 5% referral rate is presumed based on the Chagas Biovigilance Network data [18]. Participants further completed a detailed questionnaire assessing demographics, lifetime infection risk factors, and personal experience after the point of blood center deferral and *T. cruzi*-positive screening notification. Survey responses and common themes arising from patient experience are the focus of this manuscript, highlighting major gaps in the healthcare needs of participant’s screening positive for *T. cruzi* and other rare diseases through routine blood donation.

## Results

The majority of study participants were born in Mexico, followed by the USA and El Salvador, with most infections likely foreign acquired (Table 1). Participant age ranged from 20 to 78 years with a median age of 52. Females and males were equally represented, and most participating donors were Hispanic. Participants resided across California (*N* = 37) and Arizona (*N* = 9). Notably, 90% of donors had never heard of Chagas disease prior to their blood center deferral. Lifetime occupational risk factors for infection included prior outdoor (*N* = 27) or agricultural (*N* = 21) work, and history of military service (*N* = 5). Many reported having lived in housing with adobe or mud walls (*N* = 29) or with palm roofs (*N* = 16) in their home countries. Eleven participants reported being bitten by kissing bugs with reactions including redness, swelling, and itching at the site of the bite. One participant reported bites on three separate occasions, the last resulting in a trip to the emergency department after itching, tingling of hands and feet, and swelling of eyes and lips.

**Table 1:** Demographics and reported clinical management following deferral letter of the forty- six donors interviewed for this study

Country of Origin, mean years living in US if foreign-born	Number (% of study population)
Mexico	23 (50)
United States	11 (24)
El Salvador	8 (17)
Argentina	2 (4)
Bolivia	1 (2)
Guatemala	1 (2)
Infection type^˦^	Number (%)
Locally acquired	11 (24)
Foreign acquired	35 (76)
Clinical Management	Number (%)
Told physician of screening results at any point	44 (96)
Sought physician visit specifically because of deferral	17 (37)
Physician ordered confirmatory blood test*	16 (36)
Referred to ID specialist*	12 (27)
Reported physician unaware of CD*	9 (20)
Reported misinformation by physician*	8 (18)
Received treatment	6 (13)

*: Of those who told physician of positive screening result at any time

*N* = 44

Similar to previous reports of *T. cruzi*-positive blood donors in the USA, common barriers among our participant population included physicians not familiar with Chagas disease, physician misinformation, difficulty with physician ordered follow-up, lack of referral to specialist, access to diagnostic testing and/or treatment, and lack of information from their physician regarding their positive screening (Table 2). Other common themes on the side of participants included frustration with not understanding the testing process, the infection itself, and not knowing next steps for managing their infection. Six participants in our study reported being told by the physician they were likely not truly infected. Physician follow-up or confirmation testing was low, even though most patients were born in endemic areas.

**Table 2:** Select participant responses reflect blood donor experience with stigma and access to disease-competent clinical care

Q: When you first found out you tested positive for the parasite that causes Chagas disease, how did you feel?
A: “*I was worried because no one know what it was, even the doctors did not know about it.”*
Q: What has been the most difficult part about testing positive for Chagas disease?
A: “*Not knowing anything, the lack information from the doctor or support.”* A: “*Frustrating that doctors can’t tell me anything and can’t do anything for me.”*
Q: After you were told that you tested positive for the parasite that causes Chagas disease, did you go to a doctor for this issue? *If no – why not?*
A: “*I asked the doctor to test me, he said no, he would not because he did not work with that.”*
Q: How did your doctor respond when you told them you told them you tested positive for the parasite that causes Chagas disease?
A: “*The doctor said don’t worry, I haven’t heard of this on [in] 20 years. It’s probably inactive.”* A: “*Acted like it’s not a big deal, said my health problems were unrelated.”* A: “*I don’t think you have Chagas Disease-if you did, you would be very sick and could not stand now.”* A: “*Asked if I had symptoms, other than that nothing, didn’t order any other tests.”* A: “*He advised me not to get treatment because it was worse than the disease.”*

## Discussion

**Fig. 1:**
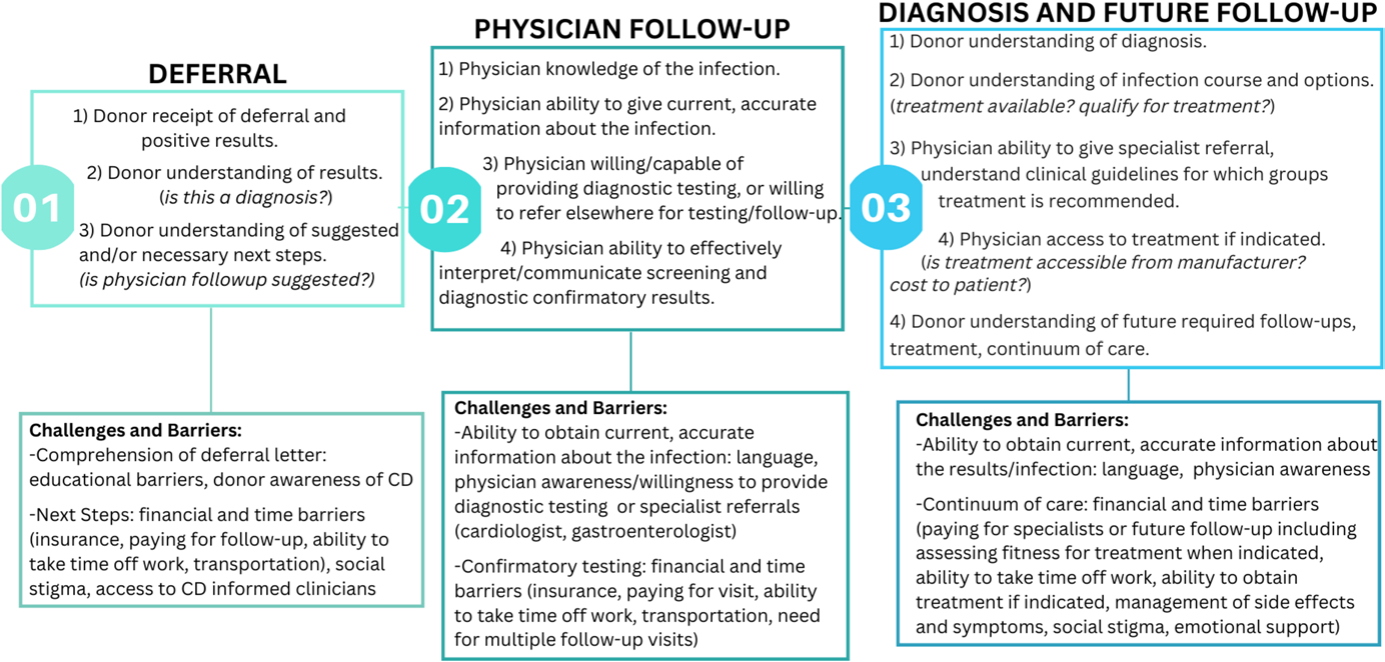
Barriers and challenges along the continuum of care for* T. cruzi*-positive screened blood donors after the point of blood donation deferral

Participant interviews highlight common challenges incurred after *T. cruzi*-positive blood donor results and deferral; themes summarized in Fig. 1. Further highlighted is the well-established need for physician awareness of Chagas and other less common transfusion-transmitted infectious diseases, as reports of physicians communicating disbelief to ultimately *T. cruzi* confirmed-positive individuals has been previously documented [19]. This expressed disbelief, coupled with misinformation and a lack of ordered follow-up diagnostic testing can create insurmountable barriers to positive donors seeking treatment. CDC guidelines strongly recommend treatment for Chagas disease for those < 50 years of age; however, only 6 of 16 confirmed-positive individuals < 50 in our study had received treatment [20, 21]. Additional barriers from this study included limited participant knowledge of Chagas disease, frustration in not understanding or having conflicting test results, and not understanding next steps to obtain clinical management after donor deferral.

The call for increased physician awareness for Chagas disease has been extensively reported, as low awareness has been frequently documented [7, 8, 22]. Prior studies have demonstrated low physician awareness, even when patients are born in endemic countries [8], posing a significant barrier to diagnosis and ultimately treatment when indicated [23]. Additionally, recent population trends indicate a shift in Latin American born populations to more rural, non-traditional regions of the USA [24–26]. Clinicians in these atypical regions, may be particularly unfamiliar with the infection, evidenced by > 80% of Ohio clinicians citing limited or very limited infection awareness [27]. Although education campaigns have proven useful, our study highlights the fact that major gaps remain, even in major metropolitan areas with historically high Latin-American born populations [28]. As blood donation screening is not sufficient as a confirmatory diagnostic tool for Chagas disease, physician ordered follow-up diagnostic testing is a critical next step in order to provide quality care to positive blood donors [29]. This secondary clinical confirmatory testing step is not unique to Chagas disease, as other blood transfusion pathogens screened among the donor supply, such as HIV and CMV, also require a secondary confirmation diagnostic prior to treatment administration.

Though much attention has been focused on increasing provider knowledge, Chagas-competent clinicians face challenges in the ability to order testing and provide follow-up care. Among recently surveyed obstetric-gynecology and family medicine practitioners who received Chagas disease education, only one-third were aware of how to order testing [28]. Clinically confirming infection is hindered by a lack of sensitive diagnostic platforms. As Chagas diagnosis requires two antigenically distinct platforms, additional testing is often necessary for a confirmatory result, and is commonly performed by CDC [4]. Only four FDA-cleared *T. cruzi* diagnostic-grade tests are available and all four have inferior diagnostic sensitivity in the USA [30]: Hemagen ELISA (Hemagen Diagnostics, Columbia, MD), InBios Chagas Detect Plus (InBios International, Seattle, WA), Ortho *T. cruzi* ELISA (Ortho Clinical Diagnostics, Inc, Raritan, NJ), and Wiener Chagatest ELISA recombinant v0.3.0 (Wiener Lab Group, Rosario, Argentina) [31]. Further certified laboratories to perform testing are also limited, and many only use one testing platform preventing the ability to perform optional diagnostic testing in the event of a false positive or false negative [31].

The public health focus on identifying and treating indeterminant Chagas disease cases in the USA is relatively new, and policies surrounding the infection are rapidly evolving. As of 2023, eight states (Arizona, Arkansas, Louisiana, Mississippi, Tennessee, Texas, Utah, Washington) listed mandatory reporting of the infection along with Los Angeles County, CA [32–35]. Due to limitations in surveillance and mandatory reportability of Chagas disease nationally, blood donor screening has become an unintentional but important method of identifying cases primarily within the research sector [12, 19, 36, 37]. As of 2020 the majority of confirmed and suspected autochthonous cases have been identified in this manner (> 2500 newly diagnosed cases) [31]. As blood center *T. cruzi* screening is often the donor’s first diagnostic test, blood centers serve as critical intervention points for improving long-term clinical management among those screening positive. FDA guidance (21 CFR 610.40d1-3[38]) states that repeat reactive donors and donors with indeterminate supplemental test results be permanently deferred from donation and notified of all testing results within 8 weeks of deferral decision[38, 39]. Recommendations also state that permanently deferred donors “be informed of the likelihood and medical significance of infection with *T. cruzi*” [38]. As noted by the donor interviews, the current deferral letters adequately inform participants of their tests results (96% of interviewed donors eventually told their physician of their Chagas disease donor deferral letter), although they fail to motivate newly diagnosed donors to actively seek clinical care (only one-third sought clinical care following receipt of their donor deferral letter) (Table 1).

Improved and standardized blood center deferral letters could prove useful in providing both donor and provider with current clinical information about the disease. Some donation center donor deferral letters are relatively comprehensive, offering explanation of *T. cruzi* testing results, suggestion for physician follow-up, and an additional letter targeted towards physicians. However, other blood center deferral letters contained outdated information, inconsistent with the current understanding of disease, high-risk groups, and treatment approvals. Approximately 143 different community- and hospital-based blood donor organizations exist nationally [40], creating a large variation in donor deferral letter content. Organizations range in size and capacity, with most organizations being very small and servicing a single metropolitan area—these centers are potentially in greater need of improved deferral letter improvements based on low staff capacity and potentially narrow medical director knowledge. Therefore, it is critical that these letters be standardized and routinely updated to ensure that accurate and current information regarding *T. cruzi* infection risk, diagnosis, and testing is communicated to both donors and physicians. Standardizing blood donor deferral letters via a regularly updated template provided by the AABB with support from the CDC and FDA could be utilized to provide deferred donors with Chagas-competent physicians and clinical institutions that are equipped to provide consultation.

**Fig. 2:**
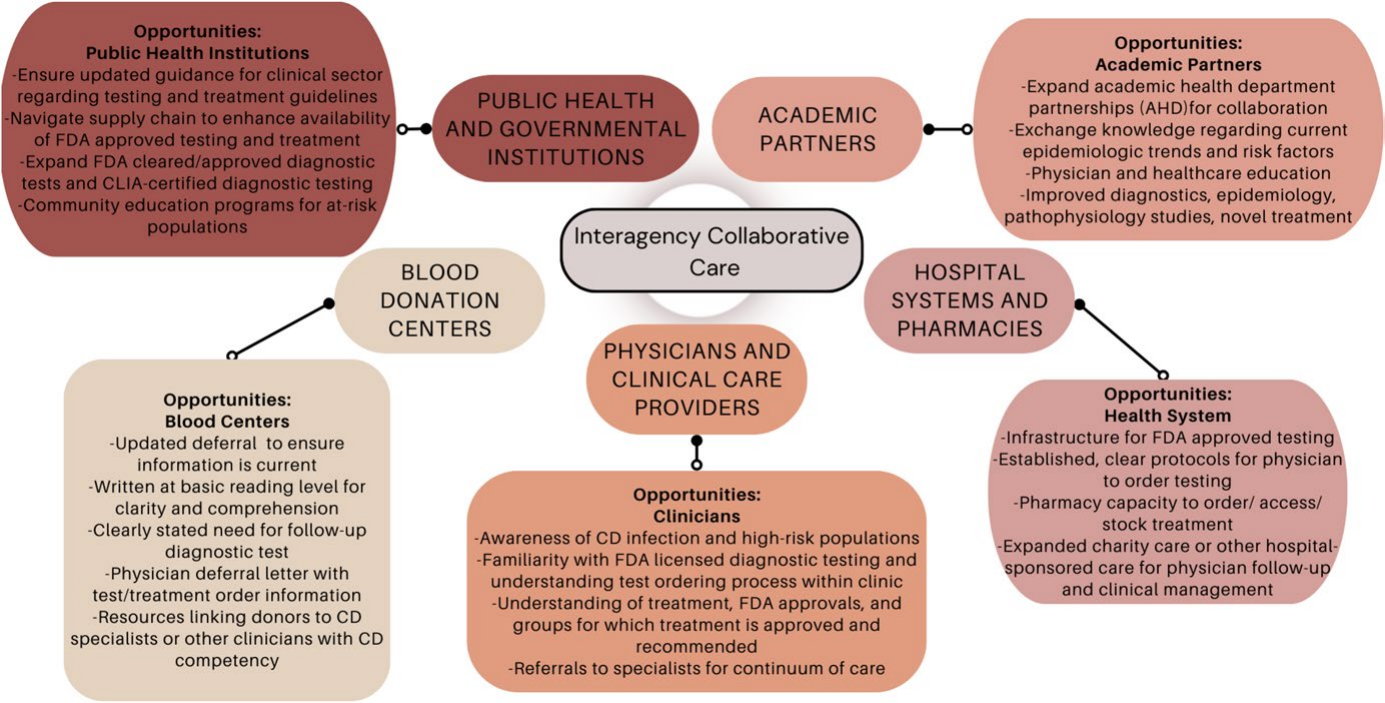
Potential points of intervention after the point of blood donor deferral to reduce challenges and barriers to diagnosis and clinical disease management of Chagas disease and other rare transfusion-transmitted infections

In addition to blood centers, public health professionals and government health agencies could support positive blood donors through increased physician and high-risk population education campaigns. Starting in 2015, the CDC began a physician education grant campaign for neglected parasitic infections, including Chagas disease. Utilization of a telemedicine-based learning course that incorporated both clinical, veterinary, and public health experts demonstrated a statistically significant increase in provider’s knowledge of Chagas disease transmission, clinical presentation, diagnostics and treatment [41]—highlighting the ability to effectively target and engage providers living in the states with the greatest number of Chagas disease donors. Alternatively, education of at-risk populations is critical to empower and motivate identified donors to seek clinical care. Individuals living with Chagas disease in the USA may be uninsured, and providers have cited challenges in continuing care for patients unable to pay for multiple follow-ups, referrals, or hospitalization [16]. Social stigma and documentation status of foreign-born persons may further provide a barrier to accessing care and completing follow-up. Therefore, employing trusted community resources for at-risk foreign-borne populations is critical to effectively educate and provide practical information for those seeking clinical management [42]. While many of these challenges will take significant time and resources to address, there is tangible opportunity for public health action for several of these identified obstacles. We highlight several of these tangible steps in Fig. 2.

## Policy implications

Despite the challenges and opportunities presented in this manuscript, questions remain as to: (1) how to connect positive donors to follow-up care, confirmatory testing, and disease management, and (2) which institutions are best equipped to handle this critical step, as well as who is responsible for connecting positive donors to clinical care?

While blood centers are often the first to identify this rare infection through routine donation, the primary responsibility of blood centers is to ensure the safety of the blood product. As many blood centers are small, community-based agencies with limited resources, further action realistically falls outside of the blood centers’ scope. The next logical organizational structure would the state health departments, however, mandatory reportability is limited nationally leaving most state health departments without the jurisdictional authority to provide clinical guidance and assistance. Further, state health departments and their public health laboratories are not equipped to test for rare pathogens, such as *T. cruzi*, and for those pathogens not on their mandatory reporting list. Local healthcare systems could expand charity programs for the uninsured to assist in incurred medical costs from seeking follow-up care. However, this would require the capacity to extend programs that are likely inaccessible for many hospital systems. Clearly an intermediary agency is warranted to serve in these capacities to connect positive donors with clinical follow-up. In all, providing positive donors with standardized deferral letters and access to clinicians and health institutions with competent Chagas disease knowledge and diagnostic capacity is both important for individual, positive donors and for the broader public health understanding of cryptic Chagas disease infection in the USA. While we highlight Chagas as the primary example in this article, these major themes could be important for other rare infections for which neither clinician awareness nor infrastructure exists, such as HTLV and babesia.

## Conclusions

For certain rare infections lacking national surveillance infrastructure, routine blood donation infectious disease screening has become an inadvertent, but useful, means of surveillance assistance. We highlight major challenges faced by individuals screening positive for transfusion-transmitted infectious diseases, with a focus on Chagas disease. There is, however, an opportunity across clinical and public health institutions to combat some of these barriers. For providers, efforts should remain focused to increase awareness. Blood centers could update and standardize deferral letters, including topically competent providers or free clinics to facilitate patient provider follow-up. Public health agencies and health institutions should focus on: addressing challenges surrounding clinical suspicion criteria, hospital system capacity to allow clinician ordered testing, hospital system expansion of charity/hospital sponsored care, availability and accessibility of emergency stock anti-parasitic treatment, awareness of treatment guidelines from USA infectious disease agencies, enhanced Chagas disease competency in medical schools, and funding for community and provider awareness campaigns to support clinicians and positive blood donors in their understanding of next steps [15, 43].

## Data Availability

Data are available from the BioStudies data repository under accession number S-BSST1367.
